# Influence of the polysaccharide capsule on virulence and fitness of *Klebsiella pneumoniae*

**DOI:** 10.3389/fmicb.2025.1450984

**Published:** 2025-02-06

**Authors:** Lisa Zierke, Rodi Mourad, Thomas P. Kohler, Mathias Müsken, Sven Hammerschmidt

**Affiliations:** ^1^Department of Molecular Genetics and Infection Biology, Interfaculty Institute for Genetics and Functional Genomics, Center for Functional Genomics of Microbes, University of Greifswald, Greifswald, Germany; ^2^Central Facility for Microscopy, Helmholtz Center for Infection Research (HZI), Braunschweig, Germany

**Keywords:** *Klebsiella pneumoniae*, capsule, adherence, phagocytosis, infection, *Galleria mellonella*

## Abstract

**Introduction:**

The capsular polysaccharide (CPS) of pathogenic bacteria is a critical virulence factor, often evading phagocytosis by host immune cells, while also interfering with the contact of the pathogen with host cells and contributing to biofilm formation. *Klebsiella pneumoniae*, a Gram-negative human pathogen associated with high antimicrobial resistances, produces 77 CPS serotypes. The CPS masks proteinaceous factors but also protects *K. pneumoniae* from uptake by host phagocytic cells and activation of the complement system. In addition to nosocomial, urinary tract and bloodstream infections or pneumonia hypervirulent strains have a highly mucoid phenotype and can cause soft tissue infections, liver abscesses, and meningitis as well. The CPS is therefore crucial for both escaping detection by the immune system and enhancing the virulence potential.

**Methods:**

In this study, we generated a non-encapsulated mutant (*Kpn*2146∆*wza*) to observe how the CPS interferes with *K. pneumoniae* adhesion, survival in blood, and invasiveness in an experimental infection model.

**Results:**

Infection of A549 lung epithelial cells showed similar adherence levels for the wild-type and non-capsulated strain, while our data showed a moderately higher internalization of *Kpn*2146Δ*wza* when compared to the wild-type. In whole blood killing assays, we demonstrate that the *K. pneumoniae* capsule is essential for survival in human blood, protecting *K. pneumoniae* against recognition and clearance by the human immune system, as well as complement-mediated opsonization and killing. The non-encapsulated mutant, in contrast, was unable to survive in either whole blood or human plasma. Infections of *Galleria mellonella* larvae showed a significantly decreased virulence potential of the CPS-deficient mutant.

**Discussion:**

In conclusion, our data indicate a crucial role of CPS *in vivo*.

## 1 Introduction

*Klebsiella pneumoniae* is a major opportunistic human pathogen responsible for a wide range of nosocomial infections and an important causative agent of healthcare- and community-acquired infections. Typical clinical manifestations are urinary tract infections, liver abscesses, bloodstream infections, and respiratory tract infections resulting in severe pneumonia (Montgomerie, 1979; Ullmann, 1998; Pastagia and Arumugam, 2008; Richelsen et al., 2020; Wang et al., 1998). The rapid spread of multidrug-resistant *K. pneumoniae* (*Kpn*) strains in recent years is a major threat to human health (Paczosa and Mecsas, 2016). Due to global spread and high morbidity and mortality rates (40–50%), carbapenemase-producing *Kpn* strains (KPC) are one of the most clinically relevant pathogens among *Enterobacterales* (Patel et al., 2008; Bowers et al., 2015; Adeolu et al., 2016; McAdam, 2020). The dominant multi-locus sequence types (ST) of KPC belong to ST258, which has emerged worldwide since the early 2000s, along with its variant ST11, the dominant strain in Asia and South America (Munoz-Price et al., 2013; Chen et al., 2014). The bacterial polysaccharide capsule (CPS) and lipopolysaccharides (LPS) are important virulence factors of Gram-negative bacterial pathogens (Holmes et al., 2021). According to their strain-specific CPS (K-antigen), *Kpn* can be differentiated into 77 capsule serotypes. To differentiate between hypervirulent and classical *Klebsiella pneumoniae* strains, various biomarkers are used. Examples include the genes *peg-344*, *iroB*, *iucA*, and *rmpA* and *rmpA2* as plasmid-based genes, which serve as biomarkers for identifying hypervirulent strains. Another biomarker of hypervirulence is the increased production of siderophores (30 μg/mL) (Harada and Doi, 2018). The CPS confers resistance to antimicrobial peptides and provides protection against recognition and uptake by professional phagocytes (Patro and Rathinavelan, 2019). Furthermore, the CPS protects against complement-mediated opsonization and killing and has been shown to modulate the host’s innate immune defense mechanisms (Simoons-Smit et al., 1986; Merino et al., 1992; Yoshida et al., 2000; March et al., 2013; Lee et al., 2014). In experimental mouse pneumonia and septicemia infection models, non-encapsulated *Kpn* strains are dramatically attenuated compared to encapsulated strains (Cortes et al., 2002; Lawlor et al., 2005; Paczosa and Mecsas, 2016). Remarkably, phagocytosed hypervirulent *Kpn* strains and other clinical strains have the capability to survive within host macrophages and neutrophils (Li et al., 2014; Cano et al., 2015). Recently, it was shown that the production of a thick capsule provides resistance against phagocytosis and provides increased virulence *in vivo* (Ernst et al., 2020).

**Figure 1:**
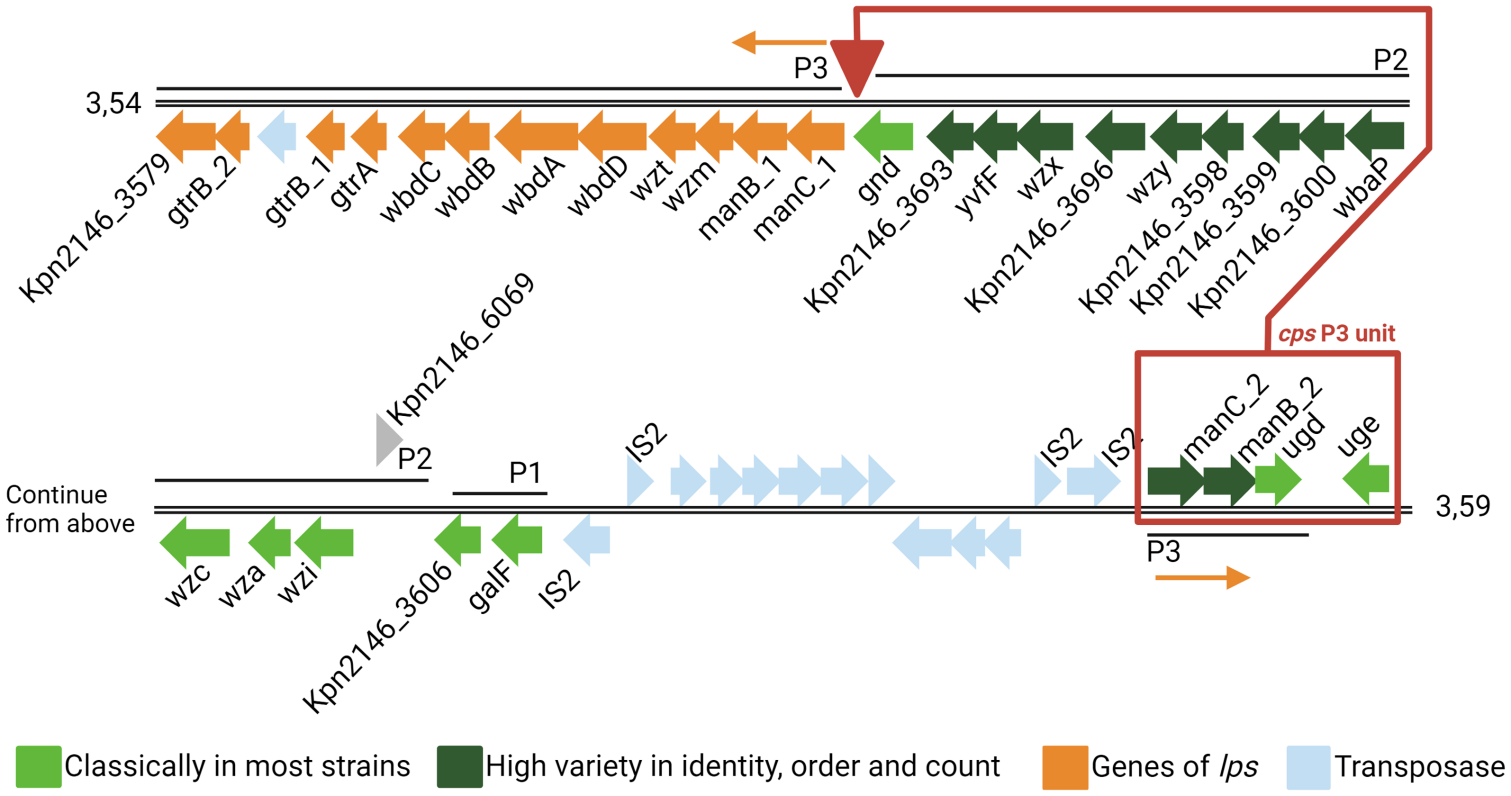
Genomic organization of the capsule locus of *Klebsiella pneumoniae* ATCC BAA2146. Coding sequences (CDSs) are represented as arrows. The color is based on the predicted functions of the resulting proteins. The three *cps* promotors P1, P2, and P3 are taken from Arakawa et al. (1995). Genes of the *cps* are in light (highly conserved) or dark green (highly variable), and genes of *lps* are in orange. Exceptionally, the *cps* P3 unit (red box) is deleted from its original position (downstream *gnd*) and inserted into the transposase region upstream of the *cps* genes cluster. The figure was adapted from Hudson et al. (2014) and NCBI GenBank CP006659.2 (Hudson et al., 2014).

The synthesis of CPS is a complex process involving various enzymes. Genes encoding the required enzymes are clustered in the so-called *cps* locus. The genes *galF*, *wzi*, *wza*, *wzc*, *gnd*, and *ugd* encoding the capsule biosynthesis enzymes of the *cps* locus are highly conserved. In contrast, genes encoding specific sugar synthesis proteins are highly variable (Pan et al., 2015). In general, the *cps* locus and the lipopolysaccharide locus (*lps* locus) are transcriptionally separated; however, strain-dependent fusion of both loci can occur. It was previously shown that in *Kpn* ATCC BAA2146 (*Kpn*2146), the fusion of the *cps-* with the *lps* locus is mediated through the deletion of the terminal *cps* P3 unit. The *lps* locus was inserted into a nearby area containing a high number of insertion sequences (Figure 1) (Shu et al., 2009; Hudson et al., 2014; Wyres et al., 2016).

**Figure 2:**
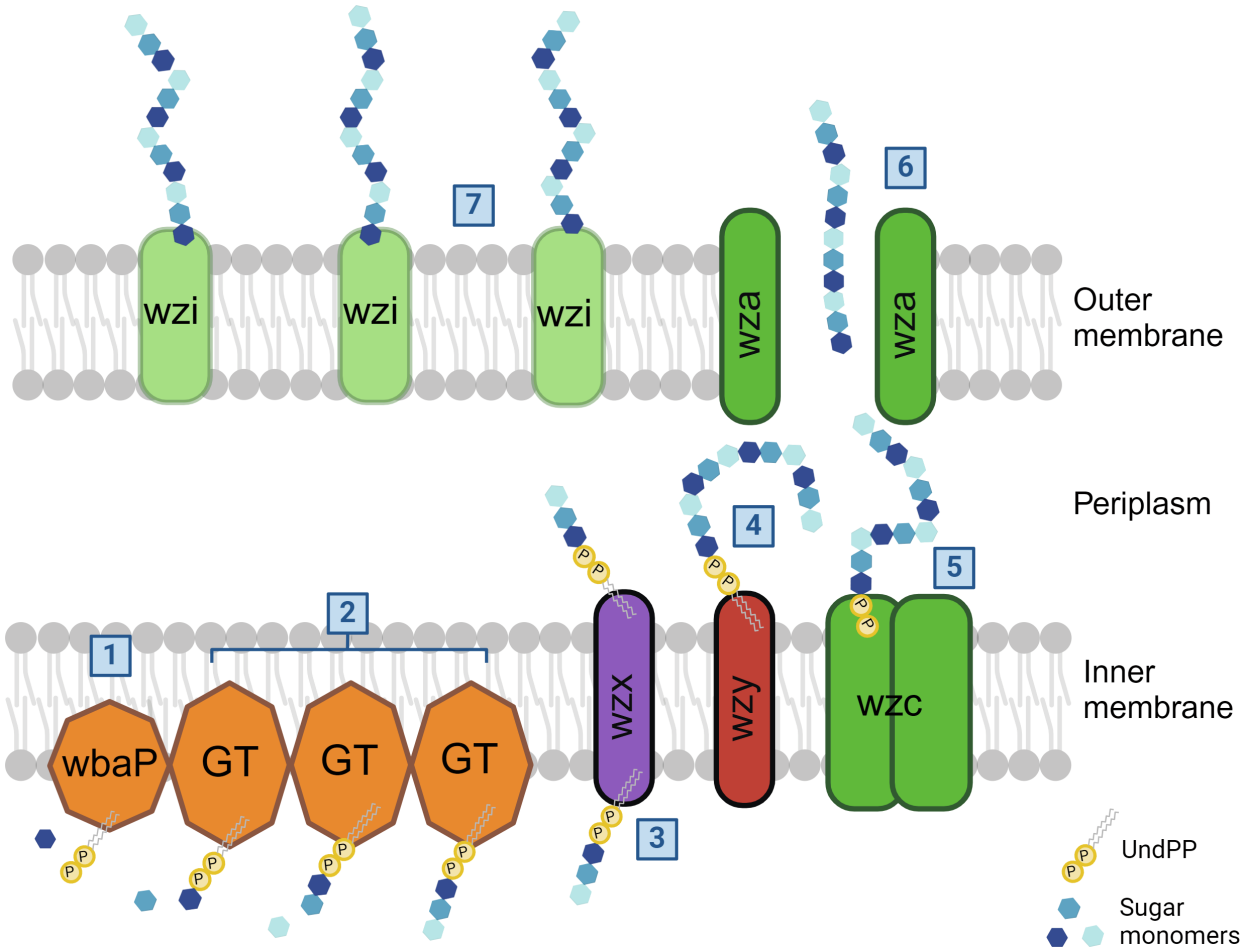
*Klebsiella pneumoniae* capsule biosynthesis pathway. In the case of *Kpn*2146, the capsule synthesis is initiated by undecaprenyl-phosphate galactose phosphotransferase WbaP by linking the first galactose to an undecaprenyl phosphate (1). Additional glycosytransferases bind additional oligosaccharides to the initial galactose (2). After flipping across the inner membrane by flippase Wzx (3), the polymerase Wzy then adds other trisaccharide units to the sugar residues (4). The length of capsule polysaccharides is then regulated by tyrosine-protein kinase Wzc (5). The outer membrane exporter Wza transfers the capsule to the cell surface (6). Wzi is then responsible for the linkage of CPS to the outer membrane (7). The figure was adapted from Olaya Rendueles, 2020 and created with BioRender (Rendueles, 2020).

The capsule production of *Kpn*2146 is, as shown in Figure 2, initiated by WbaP, a *u*ndecaprenyl-phosphate galactose phosphotransferase that links galactose to an undecaprenyl phosphate (Und-P). Other glycosyltransferases (GTs) add other oligosaccharides, which are at the non-reducing end of the glycosyl-Und-P. The flippase Wzx flips the oligosaccharide across the inner membrane into the periplasmic space where the polymerase Wzy polymerizes other trisaccharide units to the residues. The tyrosine kinase Wzc regulates the length of capsule polysaccharide chains, which are transferred to the outer membrane by the exporter Wza. In the final step, Wzi anchors the capsule to the cell surface (Whitfield, 2006; Rendueles, 2020). It has been demonstrated in *Escherichia coli* that the absence of Wza does not lead to the accumulation of capsule polymers in the periplasm, suggesting that the export of capsule polysaccharides is coupled to polymerization (Drummelsmith and Whitfield, 2000; Nesper et al., 2003).

In 2010, *Kpn*2146 was the first clinical isolate found to encode for the so-called New Delhi metallo-*β*-lactamase NDM-1, which presents a high resistance to β-lactam antibiotics in the United States and belongs to ST11 (CDC, 2013; Hudson et al., 2014). The non-profit organization American Type Culture Collection (ATCC) has conducted an extensive investigation regarding antimicrobial resistance, in which *Kpn*2146 showed resistance to all tested antimicrobial and antimicrobial inhibitor combinations including antibiotics and antimicrobial peptides (ATCC, 2024).

In this study, the highly conserved *wza* gene encoding the polysaccharide export protein was deleted in *Kpn*2146 by insertion–deletion mutagenesis. The pathophenotype of the generated non-encapsulated mutant was compared to the wild-type. We therefore assessed the phenotype and the capacity to adhere and invade in non-professional and professional phagocytes, and, importantly, we investigated the role of the CPS for survival under *in vitro* and *in vivo* conditions.

## 2 Methods

### 2.1 Ethics statement

The use of whole blood from healthy adult individuals was approved by the Ethics Committee of the University Medicine Greifswald (BB 117/23). All volunteers gave written informed consent in accordance with the Declaration of Helsinki. All experiments were carried out in accordance with the approved guidelines.

### 2.2 Bacterial strains

Bacterial strains used in this study are listed in Table 1. The cultivation of *Kpn* and *E. coli* strains was performed in lysogeny broth (LB) medium at 37°C. If necessary, LB was supplemented with 200 μg/mL chloramphenicol or 200 μg/mL spectinomycin.

**Table 1:** Bacterial strains.

Strain	Description	No.	Source
*E. coli* 10β	host of pKOV		New England Biolabs GmbH
*E. coli* DH5α	host of pGEM_spec^r^	E96	New England Biolabs GmbH
*E. coli* 10β	pMiniT::*wza*_up_down	EKp005	This study
*E. coli* 10β	pKOV::*wza*_up_down	EKp007	This study
*E. coli* 10β	pKOV-∆*wza*_specR	EKp008	This study
*K. pneumoniae* ATCC BAA2146	*Kpn*2146, wild-type		ATCC
*K. pneumoniae* ATCC BAA2146Δ*wza*	*Kpn*2146Δ*wza*, Spec^r^	KpM40	This study

### 2.3 Construction of a *K. pneumoniae* capsule-deficient mutant

To construct an isogenic capsule-deficient mutant of *Kpn*, the capsule polysaccharide exporter gene *wza* and 500 bp up- and downstream regions were amplified using primers 2187 and 2188 (see all primers in Table 2). The purified PCR product was ligated with pMiniT (all plasmids are listed in Table 3), and *E. coli* 10β chemically competent cells were transformed with the resulting plasmid pMiniT::*wza*_up_down. The purified plasmid was digested with *Not*I and *Bam*HI. Afterward, the purified DNA insert containing the *wza* gene region was ligated with similarly digested pKOV. *E. coli* DH5α chemically competent cells were transformed with the resulting plasmid pKOV::*wza*_up_down. After plasmid purification, pKOV::*wza*_up_down was used as a template for an inverse PCR reaction using primers 2189 and 2190. To replace the *wza* gene with an antibiotic resistance marker, the spectinomycin resistance gene (*aad9*) was amplified by PCR by using pGEM_spec^r^ (Table 3) and primers 2185 and 2186. The final plasmid construct for mutagenesis pKOV::Δ*wza* was used to transform competent *Kpn*2146 via electroporation. Therefore, *Kpn*2146 was incubated by shaking at 37°C until an OD_600_ of 0.5–0.7 was reached. After cell harvesting, the bacteria were washed twice with 15 mL of ice-cold 10% glycerol and finally resuspended in 1 mL of 10% ice-cold glycerol. Then, 50 μL of these electro-competent bacteria were transferred to a pre-cooled electroporation cuvette and mixed with 500 ng plasmid DNA. An electric pulse was applied with 25 μF, 200 ohms, and 1,800 V. Transformants were cultured on LB agar plates containing 200 μg/mL chloramphenicol at 37°C and 5% CO_2_, and positive clones were selected after overnight culture. For plasmid curing, positive clones were cultivated in LB medium containing 200 μg/mL spectinomycin and 5% sucrose at 40°C for 4 h. Cured clones were selected on LB agar plates containing 200 μg/mL spectinomycin at 37°C and 5% CO_2_ overnight. The successful chromosomal deletion of *wza* was verified using PCR with primers specforNheI (2185) and cpsKO_Reverse_BamHI (2188) and sequencing.

**Table 2:** Primers.

Primer	No.	Sequence 5′→ 3′	Description
cpsKO_Forwar_NotI	2,187	GCGCGCGGCCGCTAAAGATGCCA TCAACTGGTATCT	Amplification of *wza* + 500 bp upstream region
cpsKO_Reverse_BamHI	2,188	GCGCGCGGATCCGTAAATAAAGA TGTAATTGCAATAATCAACTTTCG	Amplification of *wza* + 500 bp downstream region
specforNheI	2,185	GCGCGCTAGCTTTTCGTTCGTGAA TACATG	Amplification spectinomycin resistance marker (*aad9*)
specrevSacI	2,186	GCGCGAGCTCAATTAGAATGAATA TTTCCC	Amplification spectinomycin resistance marker (*aad9*)
inversP1NheI	2,189	GCGCGCTAGCAATGTCACATCATCA GTAAACC	Inverse PCR, *wza* knock out
inversP2SacI	2,190	GCGCGAGCTCAATTATGTTCAATTCA ATTCTTGTT	Inverse PCR, *wza* knock out
wzacheckfor	2,191	CGGCATTGGCATTAGCAATAGG	Sequencing
wzacheckrev	2,192	GCCATGTCTTAATATAGCGGAC	Sequencing
pKOV3-L	2,195	AGGGCAGGGTCGTTAAATAGC	Sequencing

**Table 3:** Plasmids.

Plasmid	Description	Source
pMiniT	Linearized vector, Amp^r^, T7 promotor, blunt restriction sites, toxic mini gene, 2,588 bp	New England Biolabs
pMiniT::*wza*_up_down	Amp^r^, 4,811 bp	This study
pKOV	Cm^r^, sacB, T-sensitive pSC101 replication origin, 8,662 bp	Addgene plasmid # 25769
pGEM-spec^r^	Spec^r^	Laboratory collection
pKOV::*wza*_up_down	Cm^r^, 7,849 bp	This study
pKOV::Δ*wza_*specR	Cm^r^, Spec^r^, 7,834 bp	This study

### 2.4 Growth analysis

Growth of *Kpn*2146 and its isogenic capsule mutant *Kpn*2146Δ*wza* was analyzed in complex tryptic soy broth (TSB) and chemically defined medium (CDM), which is the non-supplemented cell culture medium RPMI-1640 (Capricorn™). Overnight cultures of *Kpn* grown on blood agar plates were used to inoculate TSB or CDM with a starting OD_600_ of 0.010 to 0.020 followed by an incubation at 37°C and 5% CO_2_. The optical density was measured hourly for 12 h, and one sample was measured after 24 h. Four biological replicates were performed.

### 2.5 Electron microscopy

To preserve the capsular polysaccharide during the EM sample preparation, bacteria were fixed with fixation solution 1 (0.15% ruthenium red dissolved in 0.2 M cacodylate buffer, 2% paraformaldehyde, 2.5% glutaraldehyde, 75 mM L-lysine) for 20 min on ice followed by two washing steps (0.15% ruthenium red dissolved in 0.2 M cacodylate buffer). The bacteria were further fixed using fixation solution 2 (0.15% ruthenium red dissolved in 0.2 M cacodylate buffer, 2% paraformaldehyde, 2.5% glutaraldehyde) for 2 h on ice and washed three times (0.15% ruthenium red dissolved in 0.2 M cacodylate buffer). The final fixation step with 1% osmium was performed at room temperature for 1 h followed by washing steps with 0.1 M EM-HEPES buffer. For SEM, samples were pipetted onto a 12 mm poly-l-lysine coated glass coverslip and fixed with glutaraldehyde. After washing with HEPES buffer, dehydration took place in a graded series of acetone (10, 30, 50, 70, 90%, and 2× 100%) for 15 min at each step. Afterward, the samples were subjected to critical point drying with liquid CO2 (CPD 300, Leica Microsystems, Wetzlar) and sputter-coated with a gold–palladium film (SCD 500, Bal-Tec, Lichtenstein). Examination was performed with a field emission scanning electron microscope FESEM Merlin (Zeiss, Oberkochen, Germany) using the Everhart-Thornley SE detector and the SE InLens detector at a 75:25 ratio, with an acceleration voltage of 5 kV. For TEM, bacterial samples were immobilized in agarose, dehydrated in a series of ethanol solutions, followed by infiltration with the resin LR White (1:1, 2:1, 100% LR White/EtOH) and polymerization was carried out at 55°C for 2 days. Ultrathin sections were generated using an Ultramicrotome Ultracut (Reichelt/Leica) and further counterstained with 4% aqueous uranyl acetate. Images were acquired using a transmission electron microscope Libra 120 (Zeiss) at an acceleration voltage of 120 kV and at calibrated magnifications.

### 2.6 Epithelial adhesion and invasion assay

Adherence to and invasion of *K. pneumoniae* into epithelial cells was analyzed using human A549 lung epithelial cells (ATCC® CCl-185™). A549 cells were cultured in DMEM (HyClone) supplemented with 10% heat-inactivated fetal calf serum (FCS, Sigma-Aldrich) at 37°C and 5% CO_2_. One day prior to the infection with *Kpn*, the A549 cells were seeded in wells of a 24-cell culture plate (Greiner Bio-One). A total of 80 to 90% confluent A549 cell layers (~1 × 10^5^ cells per well) were infected with an MOI 10 or MOI 50 of early exponentially grown wild-type and capsule-deficient bacteria in infection medium (DMEM, HyClone) in the presence of 1% heat-inactivated fetal calf serum. The infection was conducted at 37°C and 5% CO_2_ for 1 h, 2 h, or 3 h. For adhesion, infected A549 cells were washed three times with phosphate-buffered saline (PBS) to remove extracellular unbound bacteria. Afterward, the cells were lysed using DMEM containing 1% saponin. To determine the number of adherent and intracellular bacteria, the suspension was plated on Columbia blood agar. To calculate the number of recovered intracellular *Kpn*, the A549 cells were washed three times with PBS and then incubated for 1 h with an infection medium supplemented with 200 μg/mL apramycin (Biozol) to kill extracellular bacteria. Afterward, the infected A549 cells were washed again three times with PBS to remove antibiotics and lysed using DMEM containing 1% saponin (Sigma-Aldrich) to release intracellular bacteria. Colony-forming units (CFUs) of intracellular bacteria were enumerated by plating the lysed host cells containing the bacteria on Columbia blood agar. All experiments were repeated three times as duplicates. All assays were analyzed using an unpaired *t-test* (GraphPad Prism version 8).

### 2.7 Phagocytosis assay

THP-1 monocytes (ATCC® TIB-202™) were cultivated in 24-well plates (2 × 10^5^ per well) using RPMI-1640 medium supplemented with 10% heat-inactivated FCS (Sigma-Aldrich) and differentiated into macrophages using 100 ng/mL phorbol 12-myristate 13-acetate (PMA Roth) for 72 h at 37°C and 5% CO_2_. Afterward, the cells were washed with RPMI-1640 medium supplemented with 10% heat-inactivated FCS and incubated for another 24 h at 37°C and 5% CO_2_. For the phagocytosis assay, *Kpn*2146 and *Kpn*2146Δ*wza* were cultured in TSB until the early exponential phase (A_600_ = 0.7–1), then centrifuged and resuspended in the infection medium (RPMI-1640 + 1% FCS). THP-1 cells, washed with the infection medium, were infected with *Kpn*2146 or *Kpn*2146Δ*wza* (MOI 10) in 1 mL infection medium. Infections were carried out for 30 min, 60 min, and 120 min at 37°C and 5% CO_2_. After infection, THP-1 cells were washed three times with phosphate-buffered saline (PBS) and incubated for another hour in the infection medium supplemented with 200 μg/mL apramycin (Biozol) at 37°C and 5% CO_2_ to remove and kill extracellular bacteria. Finally, phagocytes were washed three times with PBS and lysed with 1% saponin (Sigma-Aldrich) to permeabilize the THP-1 cells. Recovered intracellular bacteria were plated on Columbia blood agar plates. In extended infection assays, the intracellular survival of *Kpn*2146 and *Kpn*2146Δ*wza* was analyzed. Here, the THP-1 cells were washed three times with PBS 1 h post-infection and continued to be incubated in the infection medium supplemented with 200 μg/mL apramycin for 6 h and 24 h. After 6 h or 24 h, phagocytes were washed three times with PBS, lysed with 1% saponin (Sigma-Aldrich), and suspensions plated on Columbia blood agar to determine the survival rate or number of recovered intracellular bacteria. All experiments were repeated six times as biological replicates. All assays were analyzed using an unpaired *t-test* (GraphPad Prism version 8).

### 2.8 Whole blood killing assay

Blood was collected from healthy male and female human donors from the median cubital vein using BD Vacutainer® sodium citrate tubes (BD). Until usage, the blood was stored at 37°C.

(i) *Kpn*2146 WT and *Kpn*2146∆*wza* were grown at 37°C on an orbital shaker until reaching an A_600_ of 0.7–1, harvested and resuspended in 0.9% NaCl. For the whole blood killing assay, 200 μL of citrate-anticoagulated blood was added per well in a 96-well plate. Bacteria were added to the blood with 1×10^6^ colony-forming units (cfu) in 10 μL and incubated for 10, 30, or 60 min at 37°C. To calculate the number of survived bacteria, the cfu was determined by plating a serial 10-fold dilution on blood agar plates.
(ii) For plasma replacement, 200 μL of citrate-anticoagulated blood was added per well in a 96-well plate and centrifuged for 5 min at 700×*g*. The plasma was removed, and the sedimented blood cells were resuspended with 5% or 10% pooled plasma of 21 donors (blood type AB) in 0.9% NaCl to a final volume of 200 μL. The wild-type *Kpn*2146 WT and the capsule-deficient mutant *Kpn*2146∆*wza* were added to the blood and incubated for 10, 30, or 60 min at 37°C. Bacterial survival was calculated as described above.
(iii) For plasma inactivation, pooled plasma of 21 donors (blood type AB) was heat-inactivated at 56°C for 20 min, leading to an inactivation of the complement binding capacity. Again, 200 μL of citrate-anticoagulated blood was added per well in a 96-well plate and centrifuged for 5 min at 700×*g*. The plasma was removed, and sedimented blood cells were mixed with 10% or 5% inactivated pooled plasma of 21 donors in 0.9% NaCl. Wild-type *Kpn*2146 WT and the capsule-deficient mutant *Kpn*2146∆*wza* were added to the blood containing inactivated pooled plasma and incubated for 10, 30, or 60 min at 37°C. Bacterial survival was calculated as described above.
(iv) To examine the effect of the complement in the absence of blood cells on bacterial survival, 200 μL pooled plasma and heat-inactivated plasma was added per well in a 96-well plate. For 50, 30, 10, and 5% active plasma, active plasma was mixed with heat-inactivated plasma accordingly. The wild-type *Kpn*2146 and the capsule-deficient mutant *Kpn*2146∆*wza* were added to the plasma and incubated for 10, 30, or 60 min at 37°C. Bacterial survival was calculated as described above.

### 2.9 *Galleria mellonella* infection model

*Galleria mellonella* larvae between 0.3 to 0.4 g (PROINSECTS, Minden, Germany) were kept in groups of 10 larvae at room temperature (21°C) in the dark for 1–2 days before infection. For survival analysis, *Kpn*2146 wild-type and non-encapsulated *Kpn*2146Δ*wza* were cultured in TSB medium until the early exponential phase (A_600_ = 0.7–1), centrifuged and resuspended in 0.9% sodium chloride. The larvae were infected via the intrahemocelic route by injecting 10 μL (2 × 10^6^ bacteria) into the right hind proleg of the larvae using a gastight microliter syringe (Hamilton) coupled with a repeating dispenser (Hamilton). After infection, the larvae were incubated in the dark with food at 37°C for up to 7 days. The larvae were monitored daily, and their survival was documented. Statistics were performed using the Gehan–Breslow–Wilcoxon test.

### 2.10 Statistics

Statistical analysis was performed using GraphPad Prism 8 software (La Jolla, CA, United States), with significant differences determined using the (unpaired) *t-test* in combination with the Mann–Whitney test. A *p*-value of less than 0.05 was considered statistically significant.

## 3 Results

### 3.1 Impact of the CPS on the physiology and morphology of *K. pneumoniae*

**Figure 3:**
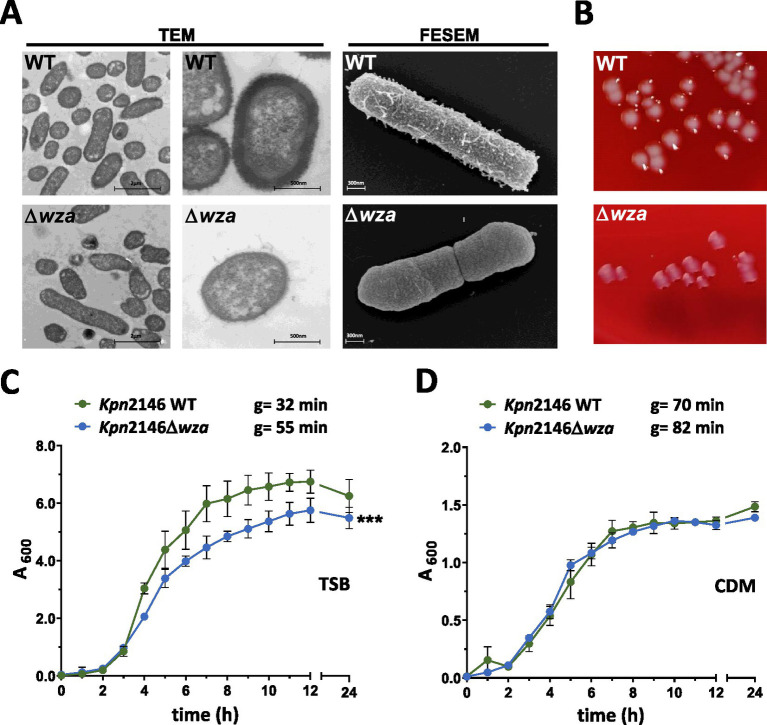
Analysis of *K. pneumoniae* cell morphology and growth behavior. **(A)** TEM and FESEM of LRR-fixed bacteria revealed a complete loss of the polysaccharide capsule. Bacteria were cultured in TSB. No difference in cell shape or cell division could be observed. **(B)** Representative colony phenotypes of *Kpn*2146 und *Kpn*2146Δ*wza* on blood agar plates (see also Supplementary Figure S1). **(C,D)** Growth of *Kpn*2146 wild-type (green) and *Kpn*2146Δ*wza* (blue) in complex TSB medium and chemically defined medium (CDM). Bacterial growth was monitored hourly for 12 h and after 24 h. g = generation time. The results are expressed as mean ± SD. Statistics: unpaired *t-test*, *n* = 4, ***p < 0.0005.

To investigate the impact of the CPS on the physiology and morphology of *Kpn* strain ATCC BAA2146 (*Kpn*2146), we have constructed a capsule-deficient strain by allelic replacement of the capsule polysaccharide exporter gene *wza*. The loss of CPS was phenotypically confirmed using electron microscopy. For both transmission electron microscopy (TEM) and field emission scanning electron microscopy (FESEM), the lysine ruthenium red fixation (LRR) method was applied to preserve the capsule of the wild-type (Fassel and Edmiston, 1999; Hammerschmidt et al., 2005). The capsule-deficient mutant *Kpn*2146∆*wza* showed a complete loss of the capsular polysaccharide material in comparison with the wild-type as indicated by TEM and FESEM (Figure 3A). The illustrations by scanning electron microscopy show the CPS in wild-type bacteria, whose colonies appear smooth on blood agar. In contrast, the capsule-deficient mutant with rough colonies on blood agar completely lacks CPS material surrounding the rod-shaped bacteria, thereby allowing the septum to be seen (Figures 3A,B; Supplementary Figure S1).

CPS production is an energy-intensive process. To evaluate the impact of the impaired CPS production on *Kpn* physiology, we analyzed the growth of *Kpn*2146 wild-type and its isogenic mutant *Kpn*2146Δ*wza* under two different physiological culture conditions. Bacterial growth was monitored in a complex medium (TSB) and a chemically defined medium (CDM) (Figures 3C,D). The generation times of the wild-type and its isogenic mutant were calculated and compared. In TSB, the capsule-negative mutant *Kpn*2146Δ*wza* showed a significantly reduced growth compared to the isogenic wild-type. The mutant reached the stationary phase at a lower optical density and showed an enhanced generation time of 52.2 min compared to 32.8 min for the wild-type. In general, *Kpn* growth in CDM is slower and shows generation times of 70.4 min for the wild-type and 82.2 min for *Kpn*2146Δ*wza*. However, the wild-type and isogenic *wza*-mutant exhibited similar growth behavior without statistically significant differences when cultured in CDM.

### 3.2 Capsule deficiency enhances internalization by human epithelial cells but not *K. pneumoniae* adhesion

**Figure 4:**
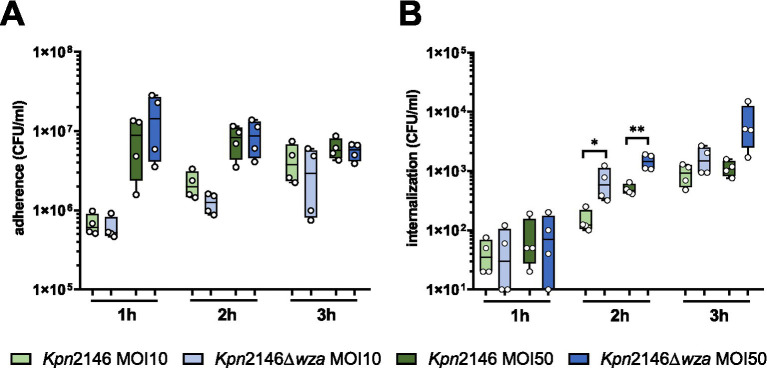
Influence of *K. pneumoniae* CPS on adherence to and internalization by human lung epithelial cells (A549). Lung cells were infected with the encapsulated *Kpn*2146 wild-type and isogenic non-encapsulated isogenic mutant *Kpn*2146∆*wza* for 1 h, 2 h, or 3 h with an MOI of 10 or 50 bacteria per lung cell. **(A)** Adherence of *K. pneumoniae* to A549 lung cells. Post-infection, colony-forming units (CFU) were enumerated by plating host cell adherent bacteria on blood agar plates after removing non-adherent bacteria. **(B)** Number of recovered intracellular *K. pneumoniae*. After infecting A549 cells, extracellular bacteria were killed at indicated time points by antibiotic treatment, host cells permeabilized, and surviving intracellular bacteria plated on blood agar plates to determine the recovered CFU of intracellular *K. pneumoniae* wild-type or CPS-deficient bacteria. Statistical analysis: unpaired *t-test*, *n* = 4, *p < 0.05, **p < 0.005.

The initial step of invasive bacterial host infections is often associated with the ability of pathogens to adhere to host epithelial or endothelial cells (Pizarro-Cerda and Cossart, 2006). We hypothesized that the CPS of *Kpn*, similar to other Gram-negative pathogens such as *Neisseria meningitidis* or *Acinetobacter baumannii*, masks adhesins or factors facilitating bacterial adherence to host cells (Hammerschmidt et al., 1996a; Craig et al., 2004; Pizarro-Cerda and Cossart, 2006; Holmes et al., 2021). We therefore investigated whether and how the CPS influences *Kpn* adherence to or invasion into human epithelial cells using the lung epithelial cell line A549. The host cells were infected with *Kpn*2146 WT or its capsule-deficient mutant *Kpn*2146Δ*wza* with an MOI 10 (multiplicity of infection) or MOI 50 for 1 h, 2 h, and 3 h, respectively. Our results showed a time-dependent increase of adherent *Kpn* irrespective of the phenotype at a low infection dose, but not when a high infection dose was used. However, at identical time points post-infection, similar numbers of host cell adherent *Kpn* were determined for wild-type and *wza*-mutant bacteria, when comparing identical MOIs (Figure 4A). Regarding uptake and internalization of *Kpn*, our data demonstrate a time-dependent increase in intracellular bacteria during infection. After 2 h and 3 h incubation of A549 cells with a low infection dose (MOI 10), the number of recovered intracellular bacteria was significantly higher for the capsule-deficient mutant in comparison with the wild-type. When using a high infection dose (MOI 50), no significant differences in recovered intracellular bacteria were observed at time points 1 h or 3 h post-infection, whereas there was a significant difference 2 h post-infection of A549 cells (Figure 4B). These results suggest that the CPS of *Kpn* plays only a minor role in adherence to lung epithelial cells but is probably important to prevent internalization by non-professional host cells.

### 3.3 The capsular polysaccharide protects against phagocytosis by macrophages

**Figure 5:**
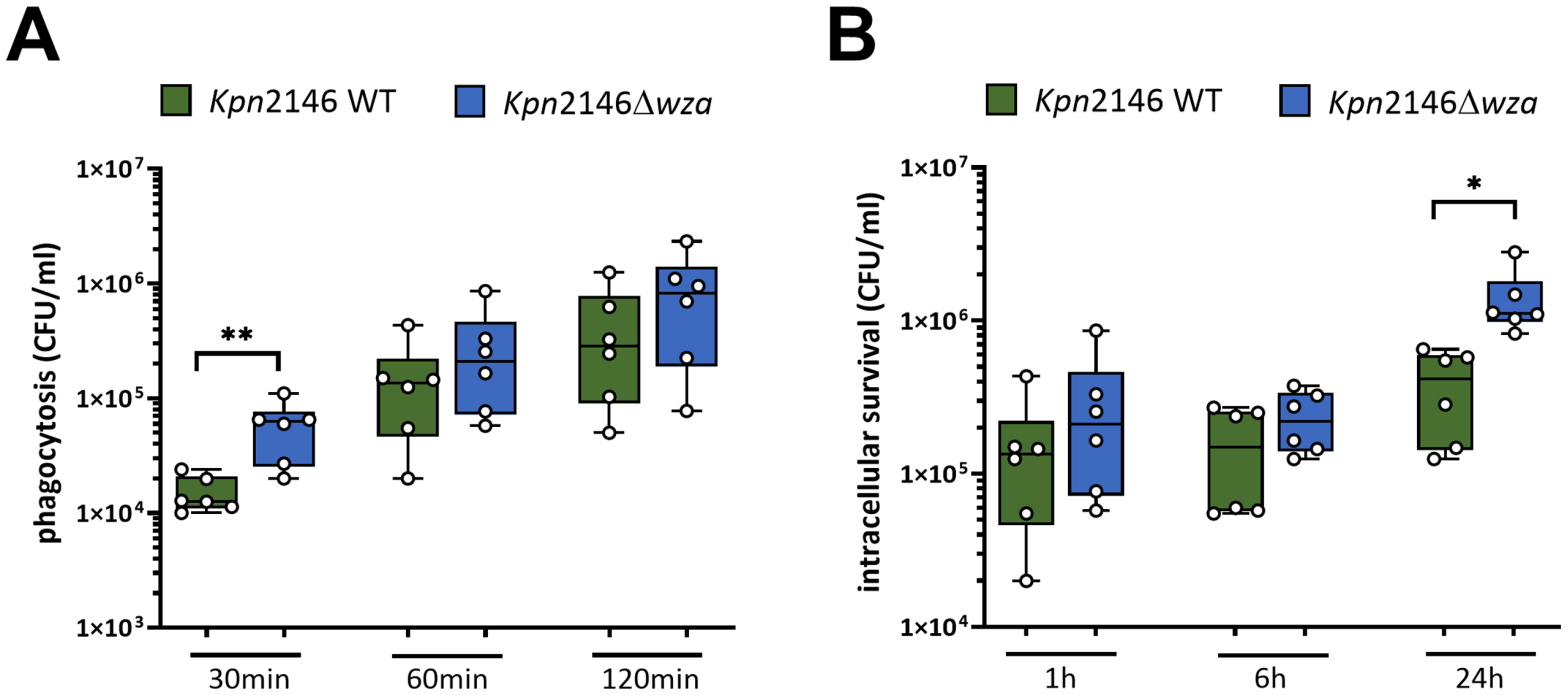
Impact of the polysaccharide capsule of *K. pneumoniae* on phagocytosis by macrophages. **(A)** PMA-differentiated THP-1 macrophages were infected with an MOI of 10 with wild-type or non-encapsulated *K. pneumoniae* for 30 min, 60 min, or 120 min. Post-infection, CFUs were determined by plating intracellular bacteria on blood agar plates. **(B)** THP-1 macrophages were infected with wild-type or non-encapsulated *K. pneumoniae* for 1 h. After 1 h, extracellular bacteria were removed by washing and further incubated in the presence of 200 μg/mL apramycin to kill remaining extracellular bacteria. After 6 h and 24 h, intracellular *K. pneumoniae* were recovered and enumerated by plating on blood agar plates. The results were presented as mean ± SD of four independent experiments. Statistical analysis: unpaired *t-test*, *n* = 6, *p < 0.05, **p < 0.005.

Next, we investigated the role of the *Kpn* CPS on phagocytosis by human THP-1 macrophages. Prior to infection with *Kpn*, THP-1 monocytes were treated with PMA (phorbol 12-myristate 13-acetate) to differentiate into mature macrophages. After infecting differentiated THP-1 cells with an MOI 10 of *Kpn* wild-type or *wza*-mutant bacteria, we determined the number of intracellular surviving bacteria. We killed extracellular bacteria with antibiotic treatment and permeabilized THP-1 macrophages containing intracellular bacteria, which were then plated on blood agar plates. Our results indicated a time-dependent uptake of both the *Kpn*2146 wild-type and its capsule-deficient mutant *Kpn*2146Δ*wza*. After 30 min of incubation, we measured a significant increase in the number of phagocytosed capsule-deficient *Kpn* compared to the wild-type (Figure 5A). When THP-1 cells were incubated for 1 h or 2 h with *Kpn*, our results did not show differences between the wild-type and the non-encapsulated *wza*-mutant (Figure 5A). We further assessed the capacity of *Kpn* to survive intracellularly. Extracellular bacteria were removed after killing with antibiotics 1 h after infection, and intracellular survivors were determined immediately, or after 6 h and 24 h, respectively. At time points 1 h and 6 h post-infection, we determined no differences between the wild-type and CPS-negative mutant (Figure 5B). Strikingly, the intracellular survival of the mutant lacking the CPS was significantly increased after 24 h in comparison with the wild-type (Figure 5B). Taken together, our data indicate a crucial role of the CPS in protecting *Kpn* against phagocytosis by macrophages at the early stage of infection. Remarkably, our data suggest that the loss of the CPS has a positive effect on the intracellular survival of *Kpn*.

### 3.4 The polysaccharide capsule protects against killing in whole blood

**Figure 6:**
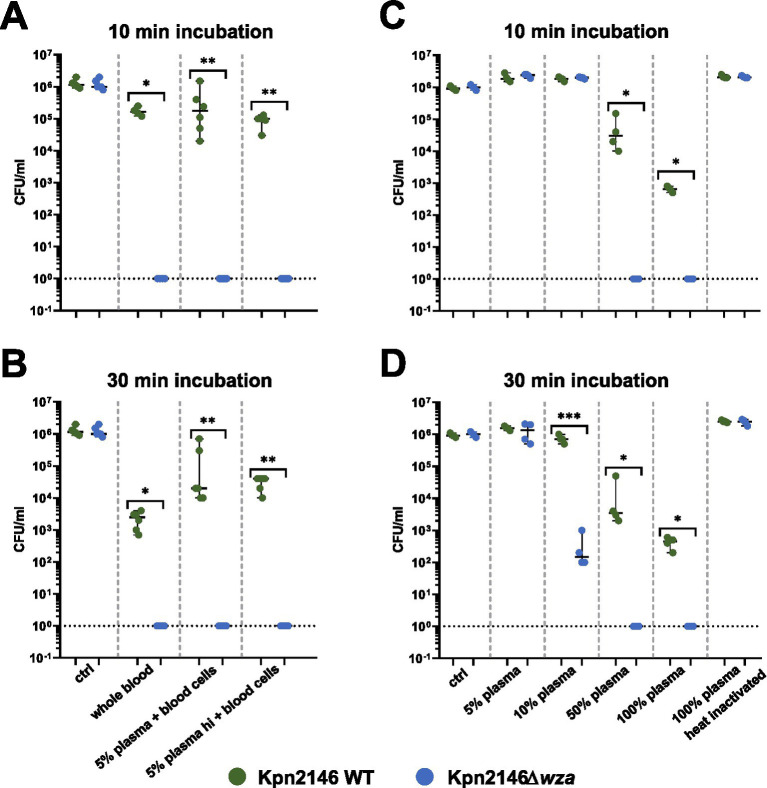
Impact of capsule deficiency on killing of *K. pneumoniae* in human (whole) blood. **(A)** Bacterial survival was determined in (i) citrate-anticoagulated whole blood (*n* = 4), (ii) blood cells with pooled 5% plasma (*n* = 6), and (iii) blood cells with pooled heat-inactivated (hi) 5% plasma (*n* = 6) in 0.9% NaCl after 10 min of incubation. **(B)** Bacterial survival was determined in (i) citrate-anticoagulated whole blood (*n* = 4), (ii) blood cells with pooled 5% plasma (*n* = 6), and (iii) blood cells with pooled heat-inactivated (hi) 5% plasma (*n* = 6) in 0.9% NaCl after 30 min. **(C)** Bacterial survival was determined with (iv) 5, 10, 50, and 100% active pooled human plasma filled with the appropriate amount of heat-inactivated plasma and 100% heat-inactivated pooled human plasma after 10 min of incubation (*n* = 4). **(D)** Bacterial survival was determined with (iv) 5, 10, 50, and 100% active pooled human plasma filled with the appropriate amount of heat-inactivated plasma and 100% heat-inactivated pooled human plasma after 30 min of incubation (*n* = 4); Mann–Whitney test and an unpaired *t-test* were used for statistics. *p < 0.05; **p < 0.005; ***p < 0.0005.

The production of a bacterial CPS is one of the most important protection strategies against recognition by the host immune system. To determine the effect of the CPS on bacterial survival in human blood, a whole blood killing assay was performed. Because of the inter-individual variability of human blood plasma, we replaced the plasma of individual donors with pooled plasma, which is the mixture of plasma sampled from several (*n* = 21) healthy donors. In a further experiment, we assessed *Kpn* killing using our pooled plasma as well as heat-inactivated pooled plasma in the absence of blood cells. We incubated *Kpn*2146 wild-type or its isogenic mutant *Kpn*2146Δ*wza* in 200 μL of (i) whole human blood (without plasma replacement) or in 200 μL (ii) human blood cells with replaced pooled active and (iii) inactivated plasma for 10 or 30 min. After incubation, bacterial survival was quantified by plating wild-type and *wza*-mutant *Kpn* on blood agar plates. The results indicated a rapid clearing of the non-encapsulated *wza*-mutant in whole human blood. Similarly, we measured a rapid killing of our non-encapsulated mutant when incubating the bacteria in human blood cells with replaced 5% (in 0.9% NaCl) active and heat-inactivated plasma. Already after 10 min of incubation, no living mutant *Kpn*2146Δ*wza* could be recovered. For *Kpn* wild-type bacteria, we measured a slight drop in the number of bacteria when using whole blood, which was more pronounced after 30 min of incubation (Figures 6A,B). The effects were even stronger when using 10% human plasma as a replacement as shown in Supplementary Figure S2.

We further tested bacterial survival in plasma and in the absence of human blood cells. In pooled plasma, we observed a concentration-dependent decrease in survival for both the wild-type and the capsule-deficient mutant. After 10 min of incubation in 50% or 100% active plasma, we did not find a single *wza*-mutant grown on blood agar, whereas the growth of the wild-type bacteria was only moderately impaired. The plasma effects were even more dramatic after 30 min of incubation. We measured a reduction in survival of the capsule-deficient *Kpn* strain when using an active plasma concentration of 10%, which was not visible after 10 min incubation. For 100% heat-inactivated plasma, no decrease in survival could be observed for the wild-type as well as the non-encapsulated mutant *Kpn*2146Δ*wza* (Figures 6B,C). In conclusion, these data demonstrate that the CPS protects *Kpn* against the recognition and clearing by the human immune system and against complement-mediated opsonization and killing.

### 3.5 Capsule deficiency attenuates *K. pneumoniae* virulence in the *Galleria mellonella* infection model

**Figure 7:**
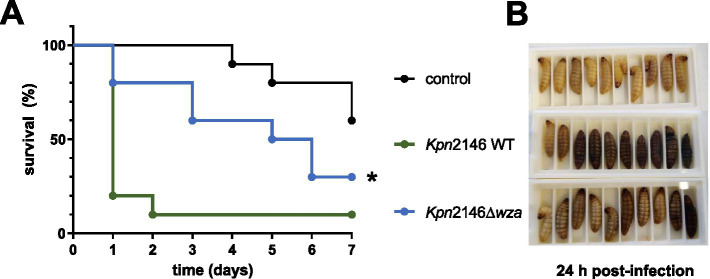
Impact of capsule deficiency on *K. pneumoniae* virulence. **(A)** Kaplan–Meier survival curve of *G. mellonella* after infection with *Kpn*2146 wild-type (green) and the capsule-deficient *Kpn*2146Δ*wza* (blue). Bacteria were grown in TSB medium to A_600_ = 0.7–1, washed with 0.9% sodium chloride, and diluted to 2 × 10^6^ bacteria per infection dose. Groups of 10 larvae (0.3–0.4 g) were infected via the intrahemocelic route and incubated for up to 7 days at 37°C with sufficient food. The survival rate was monitored daily. **(B)** Groups of larvae (Control, *Kpn*2146 WT infected and *Kpn*2146Δ*wza* infected) 24 h post-infection. All larvae of the control group were alive, and eight out of ten larvae were infected with the wild-type strain died during the first 24 h. For the capsule-deficient mutant, only two out of ten died in the first 24 h, but the infection is clearly visible. *p < 0.05.

The *G. mellonella* larvae model was applied to analyze the impact of the CPS on *Kpn* virulence *in vivo*. *G. mellonella* larvae were infected via the intrahemocelic route with *Kpn*2146 wild-type or the isogenic capsule-deficient mutant *Kpn*2146Δ*wza*. First, the infectious dose was determined (Supplementary Figure S3). Using an infection dose of 2 × 10^6^ bacteria, eight out of ten infected larvae died 24 h post-infection when infected with the wild-type (Figure 7A). In contrast, only two of the larvae infected with the capsule-deficient mutant *Kpn*2146Δ*wza* died 24 h post-infection, although the infection was clearly visible due to the darker color of the larvae (Figure 7B). Even 7 days post-infection, 30% of the larvae infected with *Kpn*2146Δ*wza* were still alive, while only 10% of wild-type-infected larvae survived. The control group showed a time-dependent death of larvae starting at day 4 post-injection of sodium chloride. This time course is comparable to that of larvae infected with *Kpn*2146Δ*wza*. Taken together, our data suggest a significant attenuation of capsule-deficient *Kpn* indicated by the decreased capability to kill *G. mellonella* larvae.

## 4 Discussion

*Klebsiella pneumoniae* is a major threat to human health due to the high number of infections and the limited therapy options (WHO, 2024). In addition to the type 1 and 3 fimbriae, siderophores, and LPS, the capsule is one of the most important virulence factors of *Kpn* (Paczosa and Mecsas, 2016). The CPS is known to protect the bacterium against killing by antibiotics and antimicrobial peptides and recognition by the host immune system (Domenico et al., 1999; March et al., 2013; Lee et al., 2014). To systematically analyze the impact of a capsule loss on bacterial fitness, pathogenicity, and survival in the host, we generated an isogenic capsule deletion mutant of *Kpn* ATCC BAA2146. The *wza*-mutant was also tested for its sensitivity against polymyxin and colistin. The results showed killing of both the wild-type and *wza*-mutant, suggesting that our mutant *Kpn*2146Δ*wza* has not undergone changes such as LPS modification or alterations in the phospholipid membrane leading to higher resistance (Supplementary Figure S4).

A prerequisite for successful bacterial colonization, which is often followed by an infection, is the ability of the pathogen to attach to non-professional host cells (Pizarro-Cerda and Cossart, 2006). It was previously shown that the bacterial CPS masks surface-associated bacterial adhesins like the self-recognizing protein antigen 43 and the type I fimbriae resulting in a reduced adhesion of wild-type bacteria to host cells in comparison with non-encapsulated mutants (Schembri et al., 2004; Schembri et al., 2005; Clements et al., 2008; Tan et al., 2020). In contrast to these studies, we found that the CPS loss does not affect the bacterial adhesion to human lung epithelial cells (A549 cells). We speculate that among others, the tip protein of the type 1 fimbriae FimH probably extending out of the CPS could be mainly responsible for the adhesion capability of *Kpn*2146 wild-type bacteria. In 2008, Stahlhut *et al.* performed an allelic diversity analysis of *fimH* in *Kpn* strains. It was assumed that a mutation in the signal peptide of *Kpn* FimH could result in the synthesis of prolonged fimbriae, which could maybe protrude beyond the CPS (Stahlhut et al., 2009). The FimH adhesion is mainly mediated via trimannosyl residues. For *E. coli*, it was found that FimH variants are also capable of mediating an additional adhesion via monomannosyl residues (Sokurenko et al., 1997). These variants are closely related to *E. coli* strains causing urinary tract infections (Sokurenko et al., 1997; Rosen et al., 2008). Because of its origin as a urinary tract isolate, *Kpn*2146 could also possess an FimH variant related to urinary *E. coli* strains. However, this is only a speculation because we have not investigated this in detail. In addition to the type 1 fimbriae, *Kpn* possesses type 3 fimbriae, which were shown to mediate adhesion to human respiratory tissues (Hornick et al., 1992; Alcantar-Curiel et al., 2013).

In some cases, the invasion of host cells is an advantage for a successful infection (Pizarro-Cerda and Cossart, 2006). The capability of *Kpn* to invade host epithelial cells was previously shown (Oelschlaeger and Tall, 1997; Sahly et al., 2000). We measured a significantly increased uptake of the capsule-deficient mutant into lung epithelial cells after 2 h of infection, indicating that the CPS could prevent the intimate contact of the pathogen with the host cells and thereby reducing the probability of *Kpn* uptake into these non-professional host cells. Ernst et al. (2020) and Kaszowska et al. (2021) described a similar effect when incubating host epithelial cells with a wild-type *Kpn* strain and an non-encapsulated deficient mutant. In contrast to our study, Ernst *et al.* used capsule-deficient mutants of clinical isolates (UCI_38, BWH_36 and BWH_45) based on the deletion of *wbaP*, the initial glycosyltransferase of capsule synthesis. For invasion assays, bladder epithelia cells were infected for 4 h, 24 h, and 48 h, and an increase in intracellular mutant bacteria was observed. In addition, Ernst et al. (2020) showed an increased persistence of capsule-deficient mutants, which resided in LAMP1-positive vacuoles. Kaszowska *et al.* also used a capsule-deficient mutant based on a knockout *wbaP*. Here, an infection of lung epithelial cells (A549) for 2 h showed a significantly higher level of internalized mutant bacteria than wild-type bacteria, which confirms our data (Kaszowska et al., 2021). Our study would have benefited from a complemented mutant expressing Wza in trans. However, when using vector systems such as pBAD33 for complementation, we were able to clone the *wza* gene into the vector systems, but we did not obtain any in trans-complementation. Wza is an exporter and is highly hydrophobic; therefore, we hypothesize that in trans-complementation and its expression from a plasmid might have toxic effects on the cell. After successfully gaining access to the blood, e.g., by transcytosis of host cell barriers, bacterial survival in the bloodstream is essential for further spreading within the host during infection. This requires the evasion from host phagocytes such as macrophages and complement, which both play an important role in the clearance of *Kpn* (Broug-Holub et al., 1997). Our results indicate that the bacterial *Kpn* CPS plays an important role during the early stage of phagocytosis. Both encapsulated wild-type bacteria and capsule-deficient *Kpn* were able to persist within the macrophages, but strikingly we found that the loss of the capsule increased the viable amount of bacteria over time. The persistence of *Kpn* within host cells was also confirmed and verified by others (Maisonneuve et al., 2017; Grubwieser et al., 2023). *Kpn* interferes with the iron metabolism of the host cells and thus survives intracellularly (Grubwieser et al., 2023). In 2015, Cano *et al.* found an accumulation of *Kpn* within a vacuolar compartment associated with the endocytic pathway in which the downregulation of the *cps* was triggered. This study suggested that downregulation is beneficial for better survival in a nutrient-poor environment (Cano et al., 2015). Taken together, our findings suggest that the loss of the capsule is advantageous for *Kpn* survival in host macrophages.

Already in the 1980s, it was found that LPS is the main factor for protecting *Kpn* against complement-mediated killing (Tomás et al., 1986). In addition, Merino *et al.* found that the exposed LPS, e.g., through the loss of the CPS can activate the complement system. However, at the same time, resistance against complement-mediated killing is provided (Merino et al., 1992). In this study, we suggest that the CPS but not LPS is a main factor for resistance against complement-mediated killing and our data are in accordance with another study (Clements et al., 2008). Clements et al. showed that both a non-encapsulated *Kpn* mutant and a double knockout, deficient for CPS and LPS, had a reduced viability in human serum after 30 min of incubation, suggesting that the CPS is necessary to prevent complement-mediated killing, while LPS is not sufficient to protect the bacteria (Clements et al., 2008).

*Galleria mellonella* had become a popular model for *in vivo* bacterial infections in the last couple of years. Here, we demonstrated that the loss of the CPS impairs the virulence of *Kpn* during host infection, further indicating the importance of the CPS under *in vivo* infections as a sine qua non-virulence factor. The wild-type was able to kill 90% of the larvae within the first 24 h post-infection, whereas only 20% of larvae died after 24 h after infection with the capsule-deficient *Kpn*. Our data are in agreement with an earlier study, showing that capsule-deficient *manC* mutants of *Kpn* exhibit reduced virulence than the encapsulated wild-type (Insua et al., 2013).

## 5 Conclusion

We demonstrated the importance of the CPS for the bacterial fitness and pathogenicity of *Kpn*. Interestingly, and in contrast to other pathogenic bacteria such as *Streptococcus pneumoniae* or *N. meningitidis* (Hammerschmidt et al., 1996b, 2005), the lack of the CPS enhances the uptake of *Kpn* into host cells. Strikingly, intracellular survival appears to be facilitated in the absence of CPS, which may link *Kpn* pathophysiology with intracellular fitness. Finally, whole blood killing assays indicated the importance of the CPS for a lower recognition of *Kpn* by blood cells and a higher complement resistance.

## Data Availability

The original contributions presented in the study are included in the article/Supplementary material, further inquiries can be directed to the corresponding author.
