# Supplementary material for: Influence of the polysaccharide capsule on virulence and fitness of Klebsiella pneumoniae
Source: Front Microbiol. 2025 Feb 6;16:1450984. doi: 10.3389/fmicb.2025.1450984 (PMC11839663; doi:10.3389/fmicb.2025.1450984)
Supplement: Supplementary file 1 [file Data_Sheet_1.pdf]

Supplementary Material

Influence of the polysaccharide capsule on virulence and fitness of *Klebsiella pneumoniae*

Lisa Zierke[1], Rodi Mourad[1], Thomas P. Kohler[1], Mathias Müsken[2], Sven Hammerschmidt[1*]

*** Correspondence:** Sven Hammerschmidt, sven.hammerschmidt@uni-greifswald.de

1 Supplementary Figures

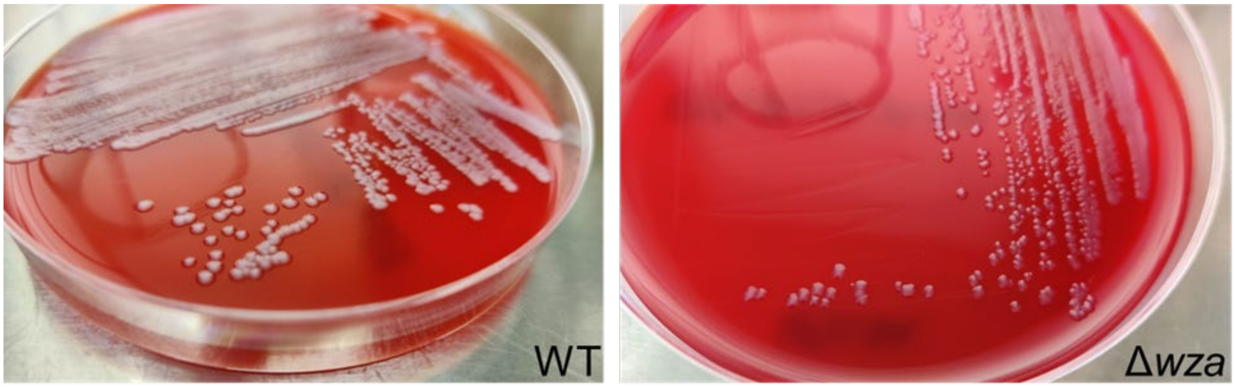

Supplementary Figure 1. Colony phenotypes of Kpn2146 wild-type (left) and the capsule deficient mutant Kpn2146Δ*wza* on blood agar plates. Wild-type colonies are larger and show a more intensive white color than the mutant colonies.

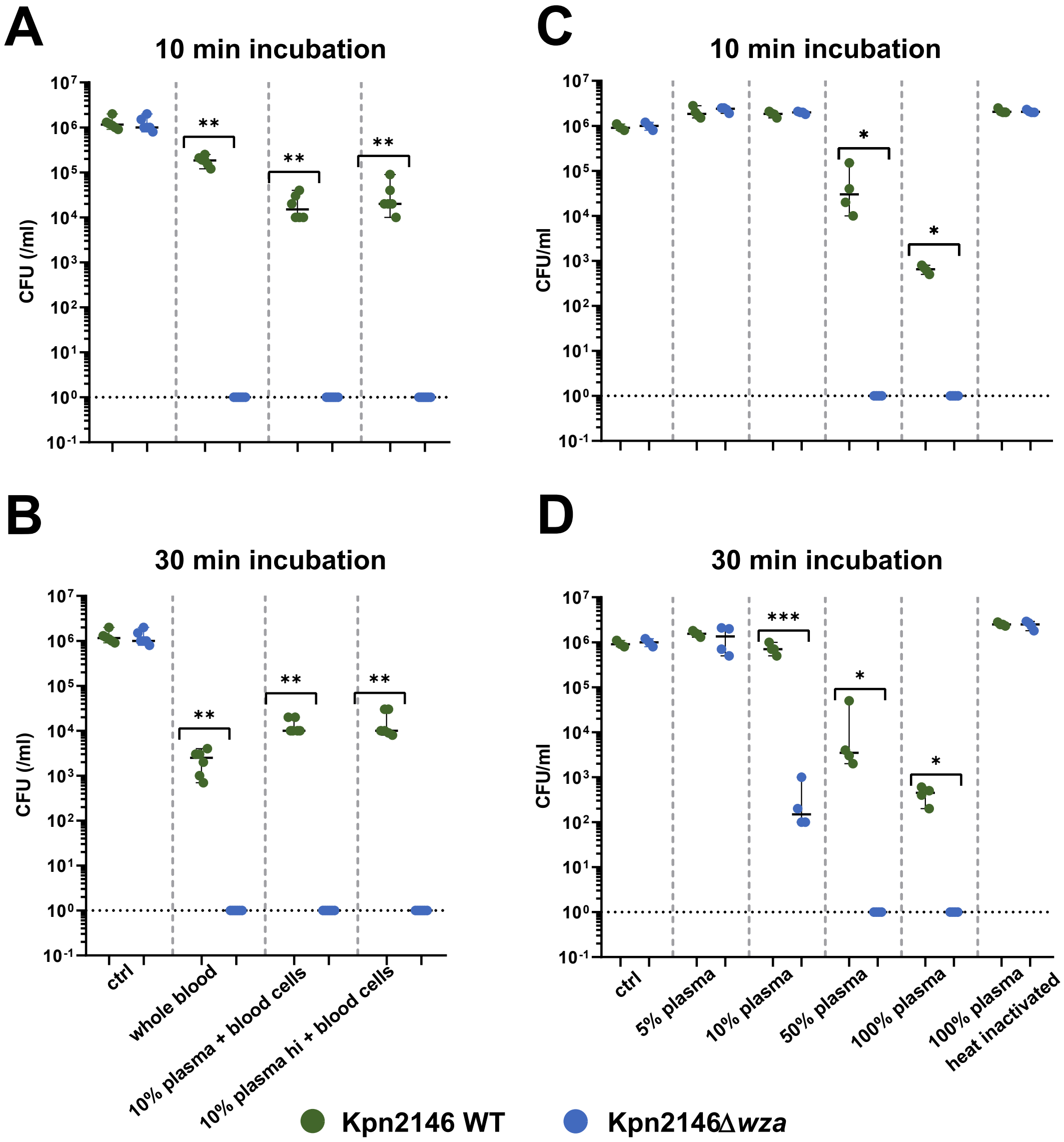

Supplementary Figure 2. Impact of capsule deficiency on killing of *K. pneumoniae* in human (whole) blood.

A Bacterial survival was determined in (i) citrate-anticoagulated whole blood (n=4), (ii) blood cells and replaced 10 % plasma (n=6), (iii) blood cells and replaced heat inactivated (hi) 10 % plasma (n=6) in 0.9 % NaCl after 10 minutes incubation. **B** Bacterial survival was determined in (i) citrate-anticoagulated whole blood (n=4), (ii) blood cells and replaced 5 % plasma (n=6), (iii) blood cells and replaced heat inactivated (hi) 5 % plasma (n=6) after 30 minutes incubation. **C** Bacterial survival was determined with (iv) 5 %, 10 %, 50 %, 100 % active pooled human plasma filled with the appropriate amount of heat inactivated plasma and 100 % heat inactivated pooled human plasma after 10 minutes incubation (n=4). **D** Bacterial survival was determined with (iv) 5 %, 10 %, 50 %, 100 % active pooled human plasma filled with the appropriate amount of heat inactivated plasma and 100 % heat inactivated pooled human plasma after 30 minutes incubation (n=4); Mann-Whitney test and an unpaired t-test was used for statistics . *p< 0.05, **p< 0.005 ***p< 0.0005

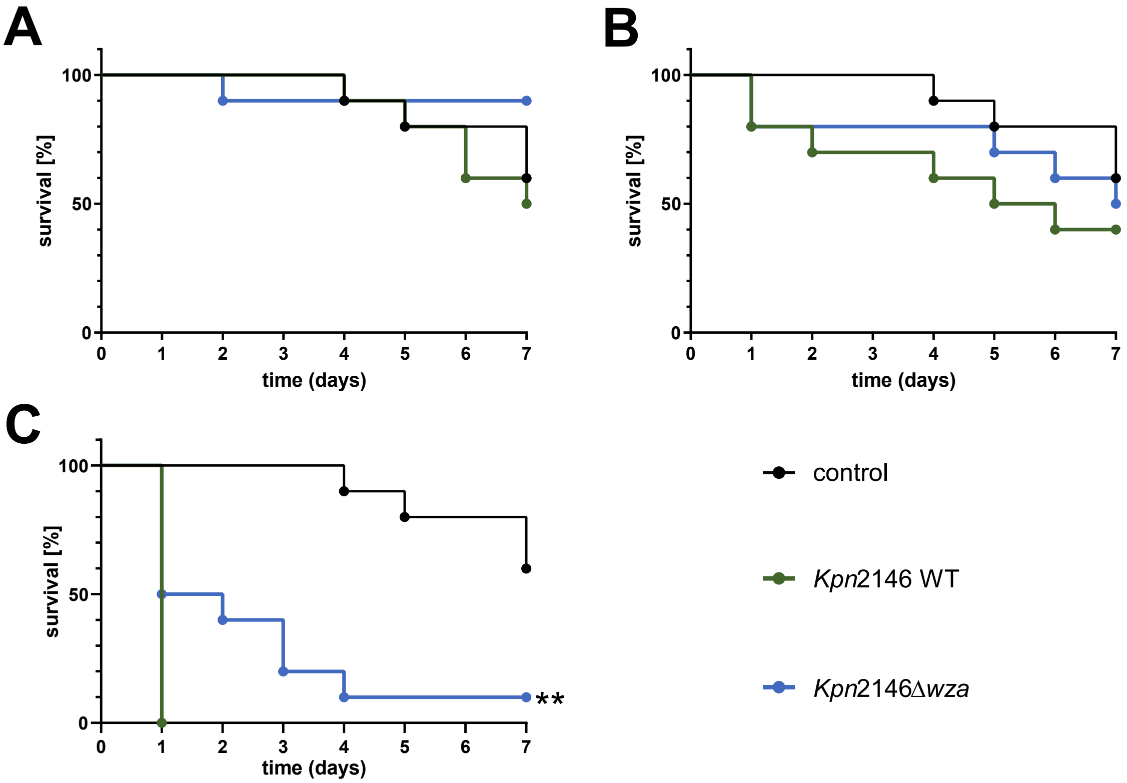

Supplementary Figure 3. Impact of capsule-deficiency on *K. pneumoniae* virulence. Kaplan Meier survival curve of *G. mellonella* after infection with *Kpn*2146 wild-type (green) and the capsule-deficient *Kpn*2146Δwza (blue). Bacteria were grown in TSB medium to A_{600} = 0.7-1, washed with 0.9 % sodium chloride, and diluted to **A** 2 x 10^4 bacteria per infection dose, **B** 2 x 10^5 bacteria per infection dose or **C** 2 x 10^7 bacteria per infection dose. Groups of 10 larvae (0.3-0.4 g) were infected via the intrahemocelic route and incubated for up to seven days at 37°C with sufficient food. The survival rate was monitored daily. *p< 0.05, **p<0,005

Colistin (10 µg)

K. pneumoniae 2146 WT

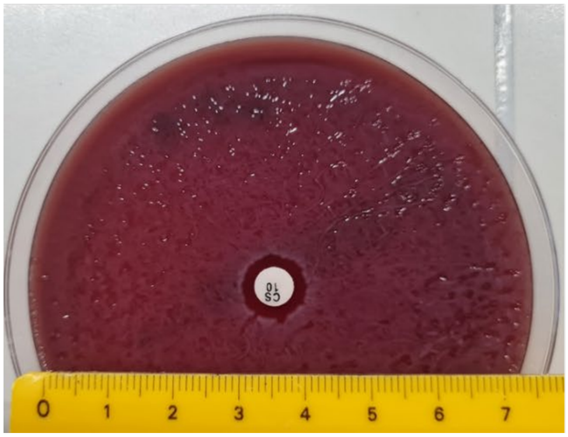

K. pneumoniae 2146Δ*wza*

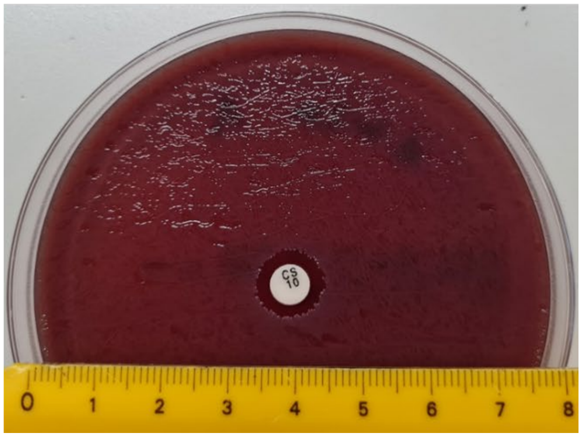

Polymyxin B (100 µg)

K. pneumoniae 2146 WT

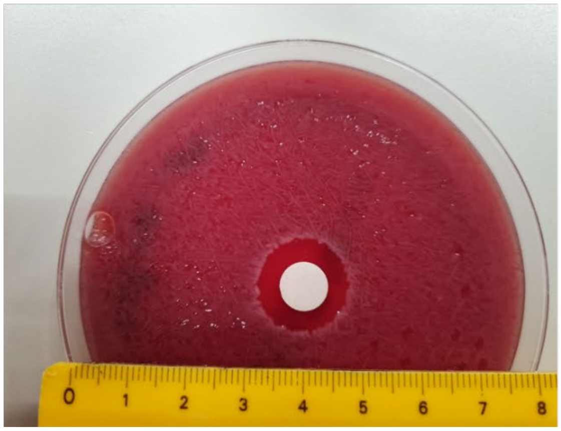

K. pneumoniae 2146Δ*wza*

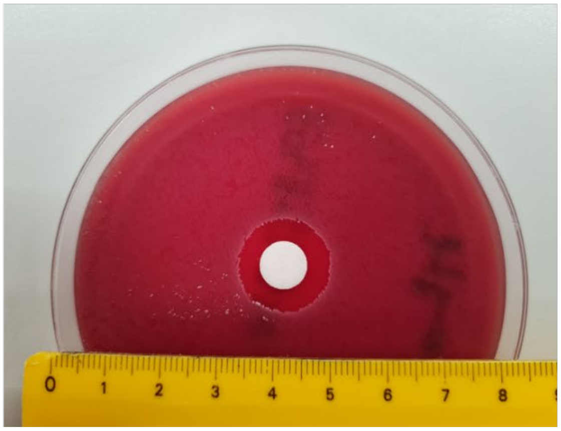

Supplementary Figure 4. Sensitivity of *K. pneumoniae* and its isogenic *Kpn*2146Δwza mutant against colistin (polymyxin E) and polymyxin B, respectively.
